# Sex-related impairment and patient needs/benefits in anogenital psoriasis: Difficult-to-communicate topics and their impact on patient-centred care

**DOI:** 10.1371/journal.pone.0235091

**Published:** 2020-07-01

**Authors:** Neuza da Silva, Matthias Augustin, Anna Langenbruch, Ulrich Mrowietz, Kristian Reich, Diamant Thaçi, Wolf-Henning Boehncke, Natalia Kirsten, Alexandra Danckworth, Rachel Sommer

**Affiliations:** 1 Institute for Health Services Research in Dermatology and Nursing (IVDP), University Medical Center Hamburg-Eppendorf (UKE), Hamburg, Germany; 2 Psoriasis-Center, Department of Dermatology, University Medical Center Schleswig-Holstein, Kiel, Germany; 3 Institute and Comprehensive Centre Inflammation Medicine, University of Lübeck, Lübeck, Germany; 4 Division of Dermatology and Venereology, Geneva University Hospitals, Geneva, Switzerland; Medizinische Universitat Graz, AUSTRIA

## Abstract

Genital psoriasis affects 2–5% of psoriasis patients; generalised plaque or intertriginous psoriasis also affects the genital area in 29–40% of cases. Anogenital psoriasis has been associated with significant quality of life impairments, but little is known about specific patient needs/treatment goals. This study aimed to examine the overall and sex-related disease burden, patient needs and treatment benefits in patients with anogenital psoriasis, compared to patients with psoriasis not affecting the anal/genital areas. Within the cross-sectional nationwide survey, 2,009 participants were consecutively recruited in 157 randomly assigned German dermatology practices and clinics, according to the following inclusion criteria aged 18 years or over; diagnosis of psoriasis vulgaris; ability to answer the questionnaires; and written informed consent. Based on a high-resolution grid on the topical distribution of psoriasis, two groups were formed: anogenital psoriasis (n = 622) and comparison group (n = 1,303). Clinical severity was assessed by the Psoriasis Area and Severity Index (PASI). Patients completed the EuroQoL visual analogue scale (EQ VAS), the Dermatology Life Quality Index (DLQI), and the Patient Benefit Index (PBI). Patients with anogenital psoriasis had higher PASI (13.0±10.6 vs. 8.9±7.6, P < 0.001) and more DLQI impairments (8.9±6.9 vs. 7.0±6.2, P = 0.002) than controls. At the item-level, they also reported more sex-related DLQI impairments (DLQI-i9: 0.5±0.8 vs. 0.3±0.7, P < 0.001) and treatment needs (PBI-i17: 2.2±1.8 vs. 1.9±1.8, P = 0.001). A great percentage of missing/not-relevant responses was found for sex-related items (23.3–41.9%). These results suggest that the assessment of sex-related impairments and treatment needs should be prioritised in patients with anogenital psoriasis. Questionnaires may be used as a less uncomfortable way for patients to discuss their genital lesions and sexual function during healthcare visits. However, the great percentage of missing/not-relevant responses to sex-related items calls for in-depth assessments and effective patient-physician communication regarding these sensitive topics.

## Introduction

The worldwide prevalence of psoriasis in adults ranges from 0.9% in the United States to 8.5% in Norway [1] and, in Germany, it affects about 2.5% of the population [2,3]. Psoriasis appears in a large variety of phenotypes which mostly result in high disease burden [4]. The prevalence of isolated genital psoriasis has been estimated between 2–5% of all patients with psoriasis. However, generalised plaque-type or intertriginous psoriasis also affects the genital area in 29–40% of psoriasis cases [5]. A recent systematic review showed significant impairments in overall and in sexual quality of life (QoL), as well as increased feelings of stigmatisation and higher rates of depression among patients with genital psoriasis [6]. Qualitative studies have also described sexual dysfunction, decreased frequency of sexual activity, avoidance of sexual relationships and worsening of symptoms after sexual activity [7,8]. The impaired sexual experience was justified by both physical effects (e.g., cracking or pain) and psychosocial effects (e.g., embarrassment and feelings of stigmatisation). Other factors that have been associated with QoL and sexual health impairments are gender, with women presenting more QoL impairments than men [6,9], and younger age [9]. Early onset of psoriasis has been also associated with higher levels of internalised stigma, which is, in turn, a predictor of decreased QoL [10].

According to the WHO Global Report on Psoriasis, one of the fundamental topics in the care of psoriasis is patient needs [11]. The wide-range variety of patient-defined therapeutic needs arise mostly from the multitude of stress and burden related to skin diseases and often diverge from the dermatologists’ treatment priorities [12]. Specifically, while the primary treatment goal for most physicians might be the objective improvement of the skin, patients report itching, scales and flaking as the most burdensome symptoms [13]. The examination of patient needs in the German and Swiss psoriasis registries (*n* = 5,343) showed that the most frequently reported needs are related to reducing physical impairments (e.g., “be healed of all skin defects”) and having confidence in therapy/healing (e.g., “have confidence in the therapy”). Of lesser importance were social goals including “to be able to have a normal sex life” or “to be able to have more contact with other people”. Significant differences in patient needs were found concerning age-group and gender [14].

However, little is known about the specific patient needs in psoriasis with anogenital involvement. On the one hand, healthcare providers do not routinely ask or examine patients for genital involvement. On the other hand, patients often feel embarrassment and discomfort about discussing these sensitive topics, which often prevents them from inquiring or from fully answer about their genital lesions or sexual (dys)function during their healthcare visits [8,15]. Specifically, about 75% of patients believe that their physician did not pay sufficient attention to their genital lesions and about 50% of patients do not discuss genital involvement with their physician [16]. As a consequence, anogenital psoriasis remains undertreated in a large portion of patients or the treatments for generalised psoriasis are contraindicated for the anogenital areas (e.g. UV therapy) [5] or they often disregard specific patient needs.

In this context, this study aimed to examine the overall and sex-related disease burden, as well as patient needs and treatment benefits in patients with anogenital psoriasis, compared to patients with psoriasis not affecting the anogenital areas. Specific objectives were: (1) to characterise general health, skin-generic QoL and treatment benefits in different groups of patients with regard to gender and anogenital involvement; (2) to examine the distribution of responses, including missing responses (MR) and not relevant responses (NRR), to sex-related items and to compare response patterns across gender and anogenital involvement groups; and (3) to estimate the associations between MR/NRR to sensitive topics and overall patient-reported outcomes (PRO) in routine care for psoriasis.

## Materials and methods

### Study design

This study is part of a cross-sectional nationwide survey in a randomly selected group of dermatology practices and clinics in Germany [17]. Out of all 3,217 office-based dermatologists in Germany with membership in the Professional Association of German Dermatologists and 119 dermatology outpatient clinics based in hospitals, 303 dermatology centres were randomly selected and asked for participation. As most patients in Germany with psoriasis are treated by dermatologists, this patient cohort represents the majority of routine healthcare for psoriasis. Of these, 157 centres agreed to participate and 126 actively recruited patients between January and August 2007.

### Participants

Patients were consecutively recruited in each centre until reaching up to 20 patients. Inclusion criteria were: (1) aged 18 years or over; (2) clinical diagnosis of psoriasis vulgaris; (3) ability to answer the questionnaires in the German language; and (4) had provided written informed consent. Patients with exclusive diagnosis of pustular or intertriginous psoriasis were excluded. Before sample collection, the study was approved by the Ethics Committee of the Hamburg Chamber of Physicians (Processing number 2767, of April 5^th^, 2007). All procedures were in accordance with the 1964 Declaration of Helsinki and its later amendments.

### Outcome measures

Standardised questionnaires were completed by the physician and by the patient. The physician questionnaire included the assessment of the clinical characteristics of psoriasis, comorbidities, and current/previous treatments. Psoriasis severity was assessed by the Psoriasis Area and Severity Index (PASI) [18] and the Body Surface Area (BSA) [19]. The patient questionnaire included the German versions of the EuroQoL visual analogue scale (EQ VAS) [20], Dermatology Life Quality Index (DLQI) [21] and Patient Benefit Index (PBI) [12]:

- The EQ VAS is a generic measure of health status and consists of a visual analogue scale ranging from 0 (“worst health”) to 100 (“best health”), using “today” as time reference.
- The DLQI is a skin-generic QoL questionnaire that includes 10 items to be answered in a 4-point Likert response scale ranging from 0 (“not at all”) to 3 (“very much”). Eight out of 10 items also include a NRR option, which is also scored as 0. A total sum score ranging from 0 to 30 was computed, with higher scores indicating more severe impairment. DLQI > 10 was considered as large/extremely large impairments on patients’ life [22].
- The PBI was validated for numerous skin diseases, including psoriasis [23] and it includes the Patient Needs Questionnaire (PNQ) and the Patient Benefits Questionnaire (PBQ), assessing, respectively, the importance of individual therapy needs and the patient-perceived benefits from treatment. Each questionnaire includes 25 items to be rated within a 5-point Likert scale ranging from 0 (“not at all”) to 4 (“very”). The option “does/did not apply to me” is also available and scored as zero for the PNQ and treated as missing for the PBQ. The PBI was computed from the arithmetic mean of all rated PBQ items weighted by the relative importance of each corresponding PNQ items, ranging from 0 (no benefit) to 4 (maximum benefit); PBI ≥ 1 was considered as having at least minimum patient-relevant treatment benefit.

In addition, the topical distribution of psoriasis was assessed by the patients, who were instructed to mark all areas/grids which they considered affected by psoriasis using a high-resolution grid scheme with 1,424 small squares [9]. For analysis, two groups were considered: anogenital psoriasis, when at least one square in the genital area (front view) or anal area (back view) was marked; comparison group, when no squares in the anogenital area were marked.

### Statistical analysis

The statistical analyses were conducted with the Statistical Package for the Social Sciences (SPSS v.23.0; IBM Corp., Armonk, NY). The level of significance was set at *P* < 0.05. Descriptive statistics (absolute/relative frequencies for categorical variables; mean and standard deviations [M±SD] for continuous variables) were obtained for sociodemographic and clinical variables. The homogeneity of sample characteristics between patients with anogenital psoriasis and controls was examined by independent-samples t-tests (continuous variables) or χ^2^ tests (categorical variables). The sociodemographic and clinical variables that significantly differed across groups were controlled in the subsequent analyses.

Differences in EQ VAS, DLQI and PBI across topology groups and gender were examined with two-way univariate analyses of covariance (ANCOVA) entering age, disease duration, PASI, BSA, and treatment as covariates. Effect-sizes were presented for the comparison analyses, considering ŋ^2^_p_ ≥ 0.01, ŋ^2^_p_ ≥ 0.06, and ŋ^2^_p_ ≥ 0.14 as small, medium, and large effects, respectively [24]. Item-level analyses were performed for sex-related QoL (DLQI-i9), patient needs (PNQ-i17) and treatment benefits (PBQ-i17). Topology and gender differences in item mean scores were tested with Mann-Whitney-U-Tests and the %MR and %NRR were compared with χ^2^ tests.

Hierarchical regression analyses were performed to examine the main and interaction effects of topology and having MR/NRR to sex-related items on general health (EQ VAS), skin-generic QoL (DLQI) and treatment benefits (PBI) [25]. After the inclusion of sociodemographic and clinical covariates in the first step of the regression equation, the independent variable, the moderator, and the interaction terms were entered as predictors in subsequent steps [26]. The strength and significance of each regression line was analysed with post-hoc simple slope computations using the Modgraph computational tool [27].

## Results

### Patient sample

A total of 2,009 patients with psoriasis were recruited (43.7% female; mean age of 51.52±14.57 years; mean age at diagnosis of 30.15±16.80 years; mean disease duration of 20.62±15.15 years; data is fully available as S1 File). Regarding clinical features, 1,739 (86.6%) patients had plaque-type psoriasis, 469 (23.3%) guttate lesions, 93 (4.6%) intertriginous psoriasis, and 31 (1.5%) pustular psoriasis. The mean PASI was 10.13±8.81, with 784 patients (39.0%) presenting moderate to severe psoriasis (PASI ≥ 10). The mean BSA was 18.07±15.84%, corresponding to 132 (6.6%) patients with mild (BSA < 3%), 588 (29.3%) with moderate (BSA between 3% and 9.9%) and 1,204 (59.9%) with severe psoriasis (BSA ≥ 10%) [20].

The study group included 622 patients with anogenital psoriasis. Regarding anogenital involvement, 317 patients (15.8%) were affected or partially affected in the genital area, 484 (24.1%) in the anal area and 179 (8.9%) presented lesions in both the genital and the anal areas. The group of patients with psoriasis involving other body areas than the anogenital (n = 1,303; 64.9%) formed the control group. The distribution of sample characteristics by topology group is displayed in Table 1.

**Table 1:** Characteristics of patients with anogenital psoriasis (n = 622) and controls (n = 1,303).

	Anogenital psoriasis	Control group	Differences		Missing
	M±SD	M±SD	t	*P*	n (%)
Age (years)	52.97±14.34	50.56±14.67	-3.39	0.001	4 (0.2%)
Age at diagnosis (years)	30.63±15.99	30.10±17.13	-0.62	0.533	153 (7.6%)
Disease duration (years)	21.82±15.03	19.66±14.96	-2.88	0.004	119 (5.9%)
PASI	12.99±10.55	8.89±7.56	-9.69	< 0.001	19 (0.9%)
BSA (%)	22.97±18.26	15.73±13.79	-9.46	< 0.001	85 (4.2%)
	n (%)	n (%)	χ^2^	*P*	n (%)
Gender			4.71	0.033	34 (1.7%)
Male	366 (58.8%)	699 (53.6%)			
Female	245 (39.4%)	581 (44.6%)			
Current therapy					
Biological therapy	84 (13.5%)	137 (10.5%)	14.12	0.046	43 (2.1%)
Conventional systemic therapy	330 (53.1%)	539 (41.4%)	26.26	< 0.001	43 (2.1%)
Topical therapy	598 (96.1%)	1,271 (97.5%)	0.09	0.778	43 (2.1%)
UV therapy	472 (75.9%)	899 (69.0%)	13.39	< 0.001	43 (2.1%)

M, mean; SD, standard deviation; t, independent samples t-test; n, number of patients; PASI, Psoriasis Area and Severity Index; BSA, Body Surface Area; χ^2^, chi-square test.

Patients with anogenital psoriasis were significantly older, had the disease for a longer time and presented higher PASI and BSA. Moreover, the study group of patients with anogenital psoriasis included more men. Regarding treatment, more patients in the study group were treated with biological, conventional systemic and/or UV therapies.

### Overall disease burden and patient benefits

Overall, the mean EQ VAS was 64.47±22.13 and the mean DLQI was 7.48±6.43. Severe QoL impairments (DLQI > 10) were presented in 630 patients (31.4%). The mean PBI was 2.49±1.14, with 1,708 patients (85.0%) presenting at least minimum patient-relevant treatment benefit. Descriptive statistics by anogenital involvement and gender, as well as comparative analyses are presented in Table 2.

**Table 2:** Univariate analyses of covariance in EQ VAS, DLQI and PBI.

		EQ VAS			DLQI			PBI		
		M±SD			M±SD			M±SD		
**Anogenital psoriasis**	Male	62.23±22.62			8.82±6.76			2.33±1.14		
	Female	59.92±24.87			9.02±7.11			2.46±1.12		
**Control group**	Male	67.71±20.43			6.50±6.04			2.49±1.11		
	Female	63.52±21.41			7.57±6.29			2.51±1.14		
		F	*P*	Ŋ^2^_p_	F	*P*	Ŋ^2^_p_	F	*P*	Ŋ^2^_p_
**Main effect of anogenital involvement**		1.60	0.207	0.001	9.27	0.002	0.006	2.03	0.154	0.001
**Main effect of gender**		13.85	< 0.001	0.009	10.22	0.001	0.006	0.21	0.646	0.000
Interaction effect anogenital involvement * gender		0.95	0.329	0.001	3.56	0.059	0.002	1.54	0.215	0.001

EQ VAS, EuroQoL visual analogue scale (0 = worst health state to 100 = best health state); DLQI, Dermatology Life Quality Index (0 = minimum impairment to 30 = maximum impairment); PBI, Patient Benefit Index (0 = no benefit to 4 = maximal benefit); M, mean; SD, standard deviation; F, two-way ANCOVA; Ŋ^2^_p,_ partial eta-square.

After controlling for age, disease duration, PASI, BSA, and type of therapy, the ANCOVAs yielded significant main effects of gender in both general health (EQ VAS) and skin-generic QoL (DLQI), with women presenting worse general health and more QoL impairments compared to men. Moreover, a significant main effect of anogenital involvement was found in the DLQI, with patients with anogenital psoriasis presenting more impairments than patients with psoriasis affecting other body areas. No significant differences according to anogenital involvement or gender were found for PBI.

### Sex-related disease burden, patient needs and treatment benefits

**Fig 1:**
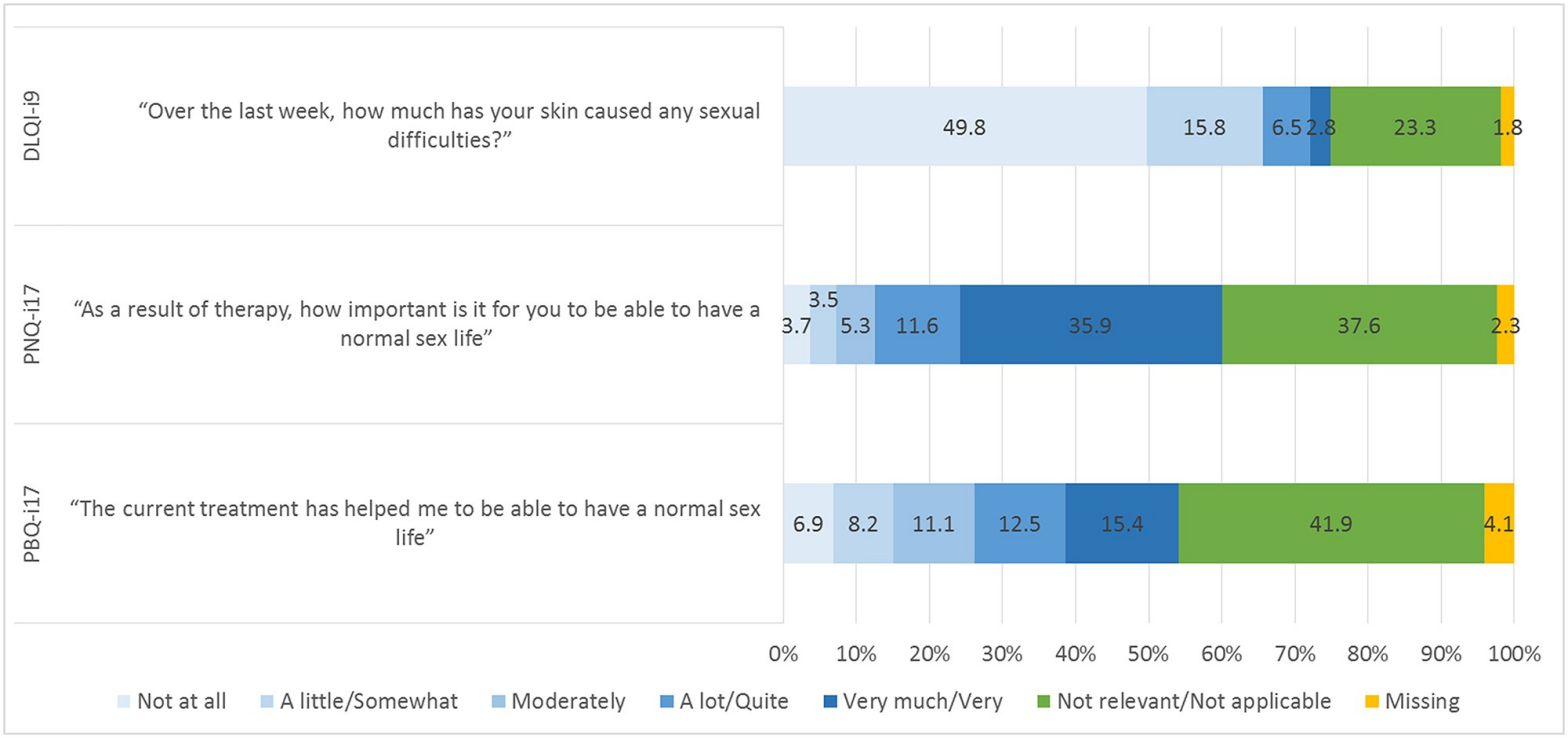
Response distribution for sex-related items. DLQI-i9, Dermatology Life Quality Index item 9; PNQ-i17, Patient Needs Questionnaire item 17; PBQi17, Patient Benefit Questionnaire item 17.

Fig 1 presents an overview of response distribution for sex-related items from the DLQI-i9 and PNQ/PBQ-i17. Regarding disease burden, the mean score for the DLQI-i9 was 0.38±0.73. However, 36 patients (1.8%) did not answer this item and 469 patients (23.3%) said it was not relevant. The overall mean score for the PNQ-i17 was 1.97±1.82 and for the PBQ-i17 was 2.39±1.37. Regarding MR, the PNQ-i17 and PBQ-i17 were not answered by 47 patients (2.3%) and 82 patients (4.1%), respectively. Moreover, to “be able to have a normal sex life” was assessed as a not applicable need by 756 patients (37.6%) and as a not applicable treatment benefit by 841 patients (41.9%).

Item-level comparative analyses for sex-related QoL impairments (DLQI-i9) and patient needs and benefits (PNQ/PBQ-i17) are presented in Table 3. For both sex-related QoL and patient needs, significant differences were found depending on anogenital involvement and gender, with patients with anogenital psoriasis and men reporting more QoL impairments and more relevant treatment needs than patients with psoriasis not involving anogenital areas and female patients. No significant differences were found for the PBQ-i17.

**Table 3:** Item-level analyses for sex-related QoL and patient needs/benefits by anogenital involvement and gender.

	DLQI-i9			PNQ-i17			PBQ-i17		
	“Over the last week, how much has your skin caused any sexual difficulties?”			“As a result of therapy, how important is it for you to be able to have a normal sex life?”			“The current treatment has helped me to be able to have a normal sex life.”		
**Scores**	M±SD	Z	*P*	M±SD	Z	*P*	M±SD	Z	*P*
Anogenital psoriasis	0.51±0.83	-5.29	<0.001	2.15±1.80	-3.19	0.001	2.28±1.41	-1.83	0.068
Control group	0.32±0.68			1.86±1.82			2.45±1.34		
Male	0.41±0.73	-3.22	0.001	2.12±1.79	-3.92	<0.001	2.41±1.35	-0.17	0.864
Female	0.34±0.74			1.78±1.85			2.38±1.41		
**Missing responses**	n (%)	χ^2^	*P*	n (%)	χ^2^	*P*	n (%)	χ^2^	*P*
Anogenital psoriasis	5 (0.8%)	2.71	0.108	6 (1.0%)	5.22	0.024	18 (2.9%)	1.83	0.200
Control group	23 (1.8%)			33 (2.5%)			54 (4.1%)		
Male	16 (1.4%)	2.06	0.176	25 (2.3%)	0.18	0.766	40 (3.6%)	1.92	0.174
Female	20 (2.3%)			22 (2.5%)			42 (4.9%)		
**NR/NA responses**	n (%)	χ^2^	*P*	n (%)	χ^2^	*P*	n (%)	χ^2^	*P*
Anogenital psoriasis	152 (24.4%)	0.98	0.33	202 (32.5%)	11.79	0.001	223 (35.9%)	15.59	<0.001
Control group	292 (22.4%)			529 (40.6%)			591 (45.4%)		
Male	197 (17.7%)	45.06	<0.001	362 (32.6%)	28.16	<0.001	413 (37.2%)	24.65	<0.001
Female	265 (30.6%)			383 (44.3%)			418 (48.3%)		

DLQI-i9, Dermatology Life Quality Index item 9 (0 = not at all to 3 = very much); PNQ-i9, Patient Needs Questionnaire item 17 (0 = not at all to 4 = very); PBQ-i17, Patient Benefit Questionnaire item 17 (0 = not at all to 4 = very); M, mean; SD, standard deviation; Z, Mann-Whitney standardised statistic; n, number of patients; χ^2^, chi-square test; NR, not relevant; NA, not applicable.

Comparative analyses of responses patterns revealed no significant differences for the %MR in the DLQI-i9, but a significantly higher %NRR was found for women compared to men. Moreover, a higher percentage of MR to the PNQ-i17 was found in the control group compared to patients with anogenital psoriasis. For both sex-related treatment needs and benefits, significant differences on %NRR were found with regard to anogenital involvement and gender, with patients with psoriasis not involving the anogenital area and females being more prone to rate item 17 as “does/did not apply to me”, compared to patients with anogenital psoriasis and men.

### Impact of missing and NRR to sex-related items on overall outcomes

The results from regression analyses are displayed in Table 4. Beyond the sociodemographic and clinical covariates, which explained 10–15% of the variance in PRO, NRR to sex-related items explained an additional 1% of the variance in general health (EQ VAS) and 6% of the variance in skin-generic QoL (DLQI). Specifically, NRR to DLQI-i9 were associated with decreased general health, NRR to PNQ-i17 were associated with increased general health, and NRR to both PNQ-i17 and PBQ-i17 were associated with less QoL impairments. Conversely, MR did not contribute to explain a significant portion of the variance in any of the outcomes, even if MR to PBQ-i17 were associated with decreased general health.

**Table 4:** Regression analyses of missing and “not relevant” (NR) / “not applicable” (NA) responses to sex-related items on general health (EQ VAS), skin-generic (DLQI) and treatment benefits (PBI).

	General health (EQ VAS)		Skin-generic QoL (DLQI)		Treatment benefits (PBI)	
	ß	t	ß	t	ß	t
**Step 1: Sociodemographic and clinical covariates**	ΔR^2^ = 0.10		ΔR^2^ = 0.15		ΔR^2^ = 0.10	
	ΔF_(9, 1564)_ = 18.76***		ΔF_(9, 1635)_ = 32.32***		ΔF_(9, 1621)_ = 18.98***	
Gender ^a^	-0.11	-4.35***	0.10	4.18***	0.001	0.004
Age	-0.07	-2.59**	-0.12	-4.83***	0.13	5.19***
Disease duration	0.001	0.03	-0.05	-2.01*	0.06	2.37*
PASI	-0.37	-6.47***	0.28	5.21***	-0.38	-6.85***
BSA (%)	0.10	1.76	0.05	0.84	0.20	3.56***
Treatment (biological therapy) ^b^	-0.02	-0.88	0.02	0.92	0.08	3.19**
Treatment (conventional systemic therapy) ^b^	0.05	1.95*	-0.04	-1.64	0.09	3.20**
Treatment (UV therapy) ^b^	-0.02	-0.64	0.05	2.28*	0.03	1.03
Anogenital involvement ^c^	-0.04	-1.47	0.08	3.47**	-0.04	-1.69
**Step 2: Missing responses**	ΔR^2^ = 0.004		ΔR^2^ = 0.002		ΔR^2^ = 0.002	
	ΔF_(3, 1561)_ = 2.15		ΔF_(3, 1632)_ = 0.97		ΔF_(3, 1618)_ = 1.17	
DLQI-i9 ^b^	-0.03	-1.18	0.02	0.75	0.03	1.13
PNQ-i17 ^b^	0.01	0.53	-0.004	-0.17	0.03	1.03
PBQ-i17 ^b^	-0.05	-2.07*	0.03	1.37	0.01	0.33
**Step 3: NR/NA responses**	ΔR^2^ = 0.01		ΔR^2^ = 0.06		ΔR^2^ = 0.002	
	ΔF_(3, 1558)_ = 6.26***		ΔF_(3, 1629)_ = 41.78***		ΔF_(3, 1615)_ = 1.25	
DLQI-i9 ^b^	-0.06	-2.44*	-0.04	-1.84	-0.03	-1.07
PNQ-i17 ^b^	0.09	2.15*	-0.17	-4.35***	0.06	1.52
PBQ-i17 ^b^	0.02	0.58	-0.09	-2.19*	-0.06	-1.47
**Step 4: Interaction effects**	ΔR^2^ = 0.004		ΔR^2^ = 0.003		ΔR^2^ = 0.004	
	ΔF_(6, 1552)_ = 1.12		ΔF_(6, 1623)_ = 1.12		ΔF_(6, 1609)_ = 1.28	
Missing response to DLQI-i9 X anogenital involvement	0.02	0.64	0.02	0.57	-0.02	-0.77
Missing response to PNQ-i17 X anogenital involvement	0.02	0.96	-0.04	-1.56	-0.02	-0.70
Missing response to PBI-i17 X anogenital involvement	-0.003	-0.11	0.03	0.99	0.01	0.46
NR response to DLQI-i9 X anogenital involvement	0.07	1.88	-0.05	-1.67	0.08	2.32*
NA response to PNQ-i17 X anogenital involvement	0.05	0.83	0.01	0.12	-0.07	-1.22
NA response to PNQ-i17 X anogenital involvement	-0.10	-1.64	0.01	0.23	0.03	0.47
**Model Summary**	R^2^ = 0.12		R^2^ = 0.22		R^2^ = 0.10	
	F_(21, 1552)_ = 9.67***		F_(21, 1623)_ = 21.33***		F_(21, 1609)_ = 8.86***	

EQ VAS, EuroQoL visual analogue scale; DLQI, Dermatology Life Quality Index; PBI, Patient Benefit Index; ß, Standardised regression coefficients; t, independent samples t-test; ΔR^2^, R^2^ change; ΔF, F change; PASI, Psoriasis Area and Severity Index; BSA, Body Surface Area; PNQ, Patient Needs Questionnaire; PBQ, Patient Benefit Questionnaire; NR, not relevant; NA, not applicable.

^a^ Gender: 0 = male, 1 = female;

^b^ 0 = no, 1 = yes;

^c^ 0 = no, 1 = yes

* *P* ≤ 0.05,

** *P* ≤ 0.01,

*** *P* ≤ 0.001, two-tailed

The non-significant interaction effects showed that the impact of MR/NRR to sex-related items on PRO was similar across patients with anogenital psoriasis and patients with psoriasis affecting other body areas. The only exception was observed for the interaction effect between NRR to DLQI-i9 and topology group: for the control group, NRR were associated with decreased treatment benefits (simple slope: b±SE = -0.19±0.08, t_(1627)_ = -2.32, *P* = 0.021), while for patients with anogenital psoriasis, the positive association between NRR responses and PBI was not statistically significant (simple slope: b±SE = 0.15±0.12, t_(1627)_ = 1.23, *P* = 0.22).

## Discussion

The present study was conducted to gain insight into the overall and sex-related QoL impairments, patient needs and treatment benefits in patients affected by anogenital psoriasis, compared to a control group of patients with psoriasis not affecting the anal or genital body regions. There is a remarkable lack of literature on disease burden in the presence of psoriasis lesions in the anal/genital skin, particularly studies relying on PRO [6]. This study has several strengths and provides innovative analyses and results, with relevant contributions for further research and clinical practice, namely: (1) the inclusion of both anal and genital psoriasis; (2) the use of a high-resolution grid scheme on the topology of psoriasis; (3) the analysis of overall and sex-related patient-defined needs and benefits from treatment in different groups in relation to gender and anogenital involvement; and (4) the examination of MR and NNR rates to sex-related items in widely used questionnaires.

Although the prevalence of exclusive genital psoriasis is estimated at 2–5% of all patients with psoriasis, 31% of patients in the current sample reported lesions in the anal and/or genital areas. The prevalence of anogenital psoriasis reported in previous studies is probably underestimated, since a large portion of patients have reported they had not been examined previously for genital involvement [28,29]. The substantial proportion of patients with anogenital psoriasis in the present study resembles those found in the few previous studies on the epidemiology of psoriasis of the genital skin [5] and may be justified by the use of a patient-reported grid scheme to document the topology of psoriasis, which enabled the patients to disclose the involvement of anogenital areas regardless of whether they have previously discussed this sensitive topic with their physicians.

Increased disease burden was found among patients with anogenital psoriasis, compared to controls. Specifically, patients with anogenital psoriasis presented longer disease duration, higher severity (PASI) and larger BSA affected, and were more frequently treated with biological, conventional systemic and/or UV therapies. They also reported more QoL impairments in general (DLQI) and in sexual functioning (DLQI-i9), as well as increased need to improve sex life (PNQ-i17). This general overview of results seems foreseeable and in accordance to previous literature [6–9], as genital psoriasis has been strongly associated with increased severity and with an increased number of previous systemic treatments (which can be considered a surrogate measure for severity over time) [30]. In addition, in-depth analyses considering patients’ gender and methodological constraints of PRO showed that the results were much more complex than the simple relationship between anogenital psoriasis and loss of QoL.

Regarding gender analyses, higher prevalence of anogenital involvement was observed in male patients; men also reported more sex-related impairments and treatment needs, at the item-level. However, they presented better health and less QoL impairments in general than women, as assessed with the EQ VAS and DLQI. These results might be explained by the higher frequency of genital itching among females, which has been described as one of the most debilitating symptoms of genital psoriasis [28,29,31], and they also highlight the limitations of index measures to detect specific QoL impairments [32]. Worse perception of body image and higher prevalence of body dysmorphic concerns among females with skin diseases in general and with psoriasis in particular may also explain the gender differences in patient-reported health and QoL [33–35].

In addition, while patients with anogenital psoriasis and males reported increased sex-related treatment needs, they did not present more treatment benefits in general or in their sex-life. This gap between patient-defined treatments needs and benefits may reflect difficulties in patient-physician communication regarding sensitive topics [8]. Specifically, the higher frequency of UV therapy among patients with anogenital psoriasis may indicate unawareness of anogenital involvement by the physicians or the disregard of anogenital involvement in clinical decisions, since UV therapy is not recommended in the anogenital area [5]. This misalignment between patients and physicians extends beyond the treatment choices, as physicians are likely to overestimate the attention paid to the patient, the time spent for the medical examination and the improvement on well-being after treatment, with deleterious effects on the patient-physician relationship, such as mistrust, seeking for “second” opinions and alternative treatments, and lower treatment adherence [36].

The missing rates were mostly negligible, invariant across gender and anogenital involvement groups, and did not contribute significantly to explain the variance in PRO. Conversely, the high portion of NRR to sex-related items in both groups of patients with and without anogenital psoriasis is worthy of further discussion. On the one hand, the great %NRR to sex-related items can be attributed to the actual absence of impact of psoriasis on sexual life (minimum impact), which is the most likely interpretation for patients without anogenital psoriasis. On the other hand, the NRR provided by patients with documented involvement of anal and/or genital areas can be attributed to the complete avoidance of social and intimate relationships because of psoriasis (maximum impact). This hypothesis is corroborated by the significant associations between more NRR to DLQI-i9 and decreased general health and between more NRR to PNQ-i17 and decreased QoL impairments. Therefore, treatment decisions based on the DLQI or even on the assessment of patient needs are potentially biased.

One important limitation that should be taken into account is the inadequacy of PASI to capture the involvement and severity of sexually sensitive areas of psoriasis [37], even if significant convergent validity between the grid scheme of topical distribution of psoriasis completed by the patient and the clinical outcomes was previously demonstrated [9]. Moreover, this study had a cross-sectional design and patient needs and benefits were assessed simultaneously with regard to current or previous treatments. No information was recorded regarding the duration of current/previous treatment or whether the involvement of sexually sensitive body areas and the specific patient needs were previously discussed in routine consultations and taken into account in treatment selection. Another important limitation was the use of data collected in 2007, as time boundaries may affect the external validity of results [38], for instance specific patient needs related to changes in social attitudes regarding psoriasis.

Despite the aforementioned limitations, the results from the present study have important implications for clinical practice and research. The remarkable portion of patients with psoriasis lesions in the anal and/or genital areas calls for routine examination of the whole skin, including the anogenital region, in daily practice. Beyond the involvement and severity of lesions in sexually sensitive body areas, the treatment choices should also take into account the sex-related impairments and patient needs regarding treatments. Patient-reported questionnaires can be first used as a less uncomfortable/embarrassing way for patients to discuss their genital lesions and sexual impairments during healthcare visits. However, it is paramount to supplement outcome assessment with in-depth qualitative interviews, including the enquiry of reasons behind NRR to sex-related items, as a basis for effective patient-physician communication and patient-centred healthcare.

Further research is also necessary to better understand the specific disease burden and treatment needs of patients with psoriasis affecting the anogenital area. Longitudinal studies addressing patient-defined sex-related treatment goals and benefits are particularly important. Additional psychosocial research in psoriasis is also imperative, mainly studies focusing on stigmatisation experiences [10], body image/dysmorphic concerns and specific mechanisms to cope with the disease, such as acceptance of oneself versus avoidance of social and intimate relationships [39] or cognitive distraction from body appearance during sexual interactions [40,41].

## Supporting information

S1 File Dataset (N = 2,009). The dataset includes anonymized data for 2,009 patients with psoriasis.
(SAV)
